# Toxic Cyanobacterial Bloom Triggers in Missisquoi Bay, Lake Champlain, as Determined by Next-Generation Sequencing and Quantitative PCR

**DOI:** 10.3390/life5021346

**Published:** 2015-05-12

**Authors:** Nathalie Fortin, Valentina Munoz-Ramos, David Bird, Benoît Lévesque, Lyle G. Whyte, Charles W. Greer

**Affiliations:** 1 National Research Council Canada, Energy, Mining and Environment, 6100 Royalmount Avenue, Montreal, QC H4P 2R2, Canada; E-Mails: nathalie.fortin@cnrc-nrc.gc.ca (N.F.); valentina.munozramos@mail.mcgill.ca (V.M.-R.); 2 Département des sciences biologiques, Faculté des sciences, Université du Québec à Montréal, Case postale 8888, Succ Centre-ville, Montreal, QC H3C 3P8, Canada; E-Mail: bird.david@uqam.ca; 3 Département de médecine sociale et preventive, Faculté de médecine, Université Laval, 945 Ave. Wolfe, Quebec City, QC G1V 5B3, Canada; E-Mail: benoit.levesque@inspq.qc.ca; 4 Institut national de santé publique du Québec, 945 Ave. Wolfe, Quebec City, QC G1V 5B3, Canada; 5 Department of Natural Resource Sciences, McGill University, 21,111 Lakeshore Road, St. Anne de Bellevue, QC, H9X 3V9, Canada; E-Mail: lyle.whyte@mcgill.ca

**Keywords:** *Microcystis*, cyanobacteria, nutrient ratio, *E. coli*, next-generation sequencing, quantitative PCR

## Abstract

Missisquoi Bay (MB) is a temperate eutrophic freshwater lake that frequently experiences toxic *Microcystis*-dominated cyanobacterial blooms. Non-point sources are responsible for the high concentrations of phosphorus and nitrogen in the bay. This study combined data from environmental parameters, *E. coli* counts, high-throughput sequencing of 16S rRNA gene amplicons, quantitative PCR (16S rRNA and *mcyD* genes) and toxin analyses to identify the main bloom-promoting factors. In 2009, nutrient concentrations correlated with *E. coli* counts, abundance of total cyanobacterial cells, *Microcystis* 16S rRNA and *mcyD* genes and intracellular microcystin. Total and dissolved phosphorus also correlated significantly with rainfall. The major cyanobacterial taxa were members of the orders *Chroococcales* and *Nostocales*. The genus *Microcystis* was the main *mcyD*-carrier and main microcystin producer. Our results suggested that increasing nutrient concentrations and total nitrogen:total phosphorus (TN:TP) ratios approaching 11:1, coupled with an increase in temperature, promoted *Microcystis*-dominated toxic blooms. Although the importance of nutrient ratios and absolute concentrations on cyanobacterial and *Microcystis* dynamics have been documented in other laboratories, an optimum TN:TP ratio for *Microcystis* dominance has not been previously observed *in situ*. This observation provides further support that nutrient ratios are an important determinant of species composition in natural phytoplankton assemblages.

## 1. Introduction

**Figure 1:**
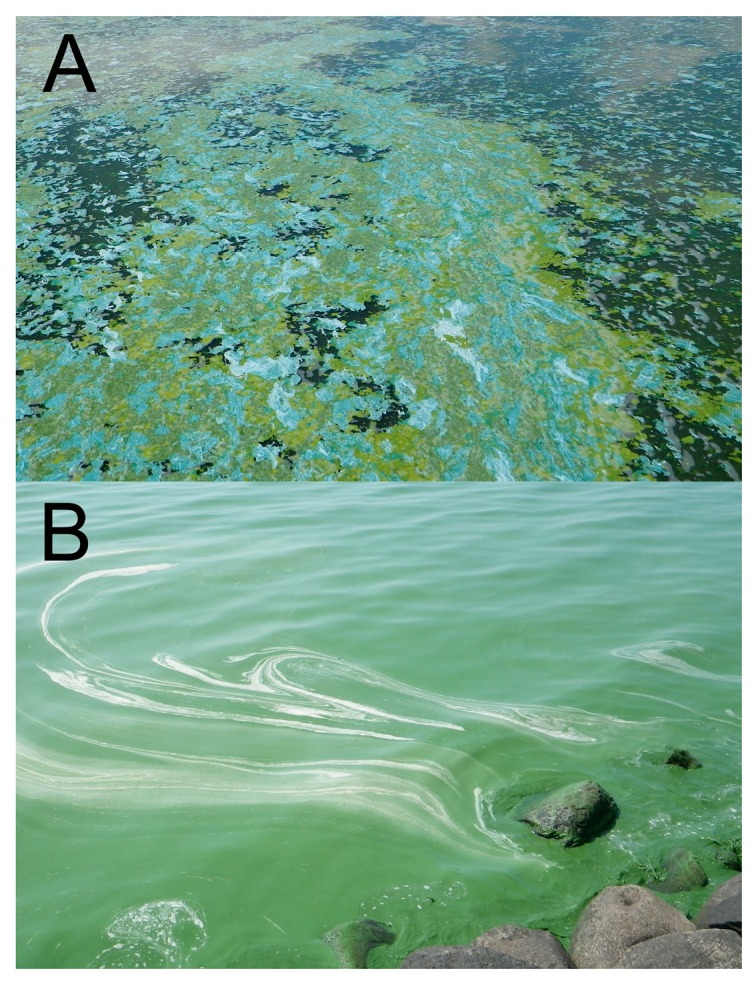
Typical cyanobacterial blooms in the pelagic area (**A**) and the littoral area (**B**) of Missisquoi Bay, Lake Champlain.

Eutrophication [1] is part of the natural evolution of aquatic ecosystems, especially of rivers, ponds, lakes and other shallow water bodies [2]. However, human activities have accelerated this process, affecting the water quality of aquatic systems and disrupting their balance, leading to the development of harmful cyanobacterial blooms (Figure 1), hypoxia and fish kills among other undesirable consequences [3,4].

Cyanobacterial blooms can negatively affect human and animal health [5,6,7,8,9,10,11], the health of the environment [3] including the contamination of potable water supplies [12,13,14] and complications in water treatment processes [12,15,16]. Indirectly, it can also impact the economy, since the resulting deterioration in water quality contributes to declines in fisheries productivity and hampers recreational activities as well as tourism [17].

Cyanobacterial blooms have been observed in freshwater, estuarine, marine environments and can develop in the photic zone [18] or at the interface between the epilimnion and metalimnion [19]. Blooms can result from the combination of a variety of bottom-up control factors, such as nutrient and light availability [20,21] and top-down control factors such as grazing and predation [22]. These, among other factors, in combination with the physical hydrological regime of the body of water, such as vertical mixing [23] and retention time [24], provide a wide set of chemical, physical and biotic interplaying variables [25], resulting in the differences in incidence, diversity and successional patterns in cyanobacterial blooms that have been observed from temperate to tropical systems worldwide [26,27,28,29,30,31].

Nutrients, such as phosphorus and nitrogen, have been identified as variables controlling phytoplankton growth and biomass [32,33]. There is general agreement that the increased incidence of cyanobacterial blooms have largely resulted from the increase in phosphorus and nitrogen concentrations from point and non-point sources [1,34]. However, it is not clear how the stoichiometric relationship between these nutrients and/or their absolute concentrations promote cyanobacterial blooms. The importance of both macronutrients on the proliferation of cyanobacteria has led to a general disagreement as to which nutrient should be controlled in order to control blooms. Management strategies have often focused on reducing P alone in order to prevent blooms [35] suggesting that the focus should be on P reduction since whole-lake enrichment experiments indicated that increasing P loads resulted in an increase of algal biomass. Schindler and colleagues suggested that controlling N inputs may in fact be counterproductive since it may result in low N:P ratios, thus promoting the proliferation of nitrogen-fixing cyanobacteria. Positive associations between P concentrations and cyanobacterial biomass have also been reported by Kotak and colleagues [36].

Management strategies to target P loading, coupled with changes in patterns of land use, increased human activities and urbanization in watersheds over the past several decades, have changed the dynamics of nutrient input into aquatic environments, resulting in N loads, sometimes at higher rates than P loads [37,38]. This increase in nitrogen loadings has sparked interest in investigating the role that N plays in the promotion of cyanobacterial blooms [22,39,40,41,42,43]. Gobler and colleagues [22] suggested that N could play an equally important role to P in bloom promotion, particularly in ones dominated by the non-nitrogen fixing genus *Microcystis*. Their field studies, coupled with nutrient amendment analyses, examined the direct effects of N on growth and toxin production. Their results indicated that N loading could promote higher growth rates of *Microcystis*, the total algal community and as well as an increase in toxin concentrations. The authors suggested that management strategies should target both N and P in order to prevent/mitigate the proliferation of all clades of toxic cyanobacterial blooms. Nutrient enrichment bioassays performed with water collected from Lake Taihu, China, revealed strong seasonal variations of nutrient limitations for phytoplankton growth [41]. During the spring and winter, the total phytoplankton biomass and growth rates increased significantly with the addition of P but not with the additions of N. However, the authors demonstrated that N was the primary limiting nutrient during the summer for the proliferation of toxic *Microcystis* blooms. Finally, a study by Davis and colleagues [42] involving nutrient amendment analyses under different temperature regimes in samples collected from four freshwater lakes in the northeast USA showed that although higher N concentrations significantly increased the *Microcystis* growth rate in some lakes, increases in temperature coupled with higher P concentrations produced the greatest growth rates in most of their experiments.

Attempts have been made to define environmental nitrogen to phosphorus ratios (as dissolved or total nutrients) that could be used in order to predict phytoplankton growth [44,45,46]. This approach has provided evidence of the influence that nutrient ratios have on the species composition of natural phytoplanktonic communities [27,46]. Smith and colleagues [46] reported cyanobacterial bloom occurrences at epilimnetic TN:TP ratios (reported as by weight) below 29:1 from 17 lakes worldwide. A boundary of TN:TP of 22:1 (reported as by weight) was identified to provide a distinct delimitation between lakes dominated by nitrogen-fixing cyanobacterial species (<22:1), from the ones where these species were rarely observed (>22:1) [47]. The cosmopolitan incidence of toxic *Microcystis* blooms has led researchers to analyze the factors that may promote dominance by this non-nitrogen fixing genus in natural assemblages. A negative correlation was observed between *Microcystis* cell abundance and the TN:TP ratio from an 11 year study of Lake Taihu, China [27]. *Microcystis* tended to dominate the phytoplankton community at TN:TP ratios below 30:1 (reported as by mass).

In contrast, Reynolds [48] argues that changes in nutrient ratios are not the promoters of the algal response. In limnetic aquatic enclosures, increases in the absolute concentration of the added nutrients, led to a decrease in the dominance of nitrogen fixing species in spite of the low nutrient ratio. The ability to predict cyanobacterial blooms based on environmental N:P ratios alone can be impaired in some ecosystems, particularly in hypereutrophic systems that have nutrient concentrations that exceed phytoplankton growth demands [25], where recycling of nutrients by biological factors maintains them at high concentrations and where favorable climatic conditions are not constant [48]. In these cases, regardless of N:P ratios, cyanobacterial growth may be periodically hindered by other factors, such as light or temperature [25,48]. This debate will clearly benefit from further studies that analyze, *in situ*, the response of toxic cyanobacterial blooms to the dynamics of phosphorus and nitrogen available in freshwaters.

The present study was conducted in Quebec, in the Missisquoi Bay (MB) area of Lake Champlain. The bay is a shallow embayment on the northern end of the lake that is separated by the international boundary between the province of Quebec and the State of Vermont. There are three major tributaries entering the bay: Missisquoi (in Vermont), Rock (Quebec and Vermont) and Pike Rivers (Quebec). The lake plays a key role in local recreational and economic activities and represents the major drinking water source for some towns and cities in the province of Quebec and in the states of Vermont and New York [49]. The bay remained mesotrophic until roughly the 1970s [50]. However, from 1979–2009, despite the fact that the total nitrogen (TN) concentrations decreased by approximately 25%, total phosphorus (TP) concentrations increased by 72%, chlorophyll a concentrations doubled, and cyanobacterial dominance increased [51]. Toxic *Microcystis* frequently dominate blooms and microcystin concentrations that exceed WHO drinking-water quality guidelines [52] have been frequently reported [18,42].

The accelerated eutrophication and resulting proliferation of cyanobacterial blooms in MB have been partly attributed to land use changes in this watershed that have resulted in increasing nutrient loadings into the bay [51]. Both Quebec and Vermont have taken steps towards reducing P loading into MB as summarized by Beck and colleagues [53]. In an effort to further control and reduce P loading into MB from point and non-point sources and to meet the guidelines of the EPA Clean Water Act, an agreement was signed in 2002, to reduce the annual in-lake TP concentrations for the euphotic zone in MB to 25 μg/L [53,54] with a total phosphorus loading capacity of 97.2 metric tons per year from the bay’s watershed. This loading capacity was divided between Vermont and Quebec sources according to a 60/40 ratio.

The pattern of land use in this watershed [55], suggests that sources of nutrients could enter the bay from run-off via agricultural lands, manure based crop fertilization or manure disposal, public campgrounds, septic systems and drain fields, wastewater treatment plants or animals. At present, point-sources account for only 4%–5% of the total P loading to MB from Quebec, while non-point sources are regarded as the main contributors to high P levels in the lake [53]. Approximately 33% [53] of the MB watershed is devoted to agricultural practices, yet 65%–79% of the total annual P load is attributed to agricultural runoff [56,57]. By 2001, 56% of the planted areas were large-scale crops, such as corn [55]. Between 1997 and 2010, the planted surface for soya has increased by 289% in the Brome Missisquoi area [58]. Both crops are highly vulnerable to erosion [59]. Livestock units (44,881) were mainly composed of pigs (49%) and cattle (39%) that generate large quantities of manure. Taking into account that the temporal distribution of precipitation intensity and snowmelt in Quebec is likely to lead to saturation-excess runoff [60], and considering the fact that fecal matter and urine are nutrient rich [61,62,63], the pattern of land use in this watershed suggests that animal excreta could be an important non-point source of nutrients entering the bay via surface runoff.

Animals are known to shed pathogens and host cells in excreta [64,65,66]. Soil and feces [66], have been identified as an important source of coliforms and *Escherichia coli* (*E. coli)* into aquatic environments [67]. It has been demonstrated that *E. coli* can survive and multiply in soils [66,68,69]. Despite the fact that *E. coli* has also been observed to be able to reproduce in water [66,67], aquatic environments are not ideal for the growth of *E. coli* [70]. Independent of their source (e.g., animal or soils), once *E. coli* cells arrive in water systems they can sink and/or be inactivated by sunlight via DNA damage, photo-oxidation of cellular components or photochemical damage [70]. It has therefore been observed that *E. coli* concentrations in water decline exponentially as a function of the time of day [70]. Based on these facts, and considering that both sources of *E. coli* (*i.e.*, feces and soils) are rich in nutrients, *E. coli* counts in surface water samples may be a potential indicator of recent nutrient input from external sources, such as surface runoff or sewage overflows. In the case of recent nutrient input from surface runoff, one would expect to find higher *E. coli* counts in the stations with greater contact with the shore and to find a correlation between *E. coli* counts and rainfall events at these stations.

In view of the multitude of stressors and responses observed from system to system during algal blooms, a site-specific characterization of the cyanobacterial taxa involved in the bloom, their potential toxicity, as well as an *in situ* analysis of the response of cyanobacterial community composition to environmental variation are essential in order to identify bloom-promoting environmental factors [71]. The objective of this project was to determine, *in situ*, the impact of physico-chemical parameters and nutrients on the dynamics of cyanobacterial community composition, abundance and toxicity in Missisquoi Bay during 2009. The selected tools included taxonomy and toxin analyses as well as quantitative PCR of the 16S rRNA and *mcyD* (involved in the synthesis of microcystin) genes. The major cyanobacterial taxa and potential microcystin producers were determined by 16S rRNA gene high-throughput sequencing using the Ion Torrent platform. We also evaluated whether *E. coli* counts could be a good indicator of recent nutrient input from surface runoff or sewage overflows.

In temperate freshwater systems, cyanobacterial blooms are frequently observed during the warmest periods of the year in nutrient rich environments [25,42]. The underlying hypothesis for this study is that once optimal temperatures are established, cyanobacterial dynamics are mainly determined by both nutrient concentrations and the TN:TP ratio in Missisquoi Bay.

## 2. Experimental Section

### 2.1. Sampling Sites

**Figure 2:**
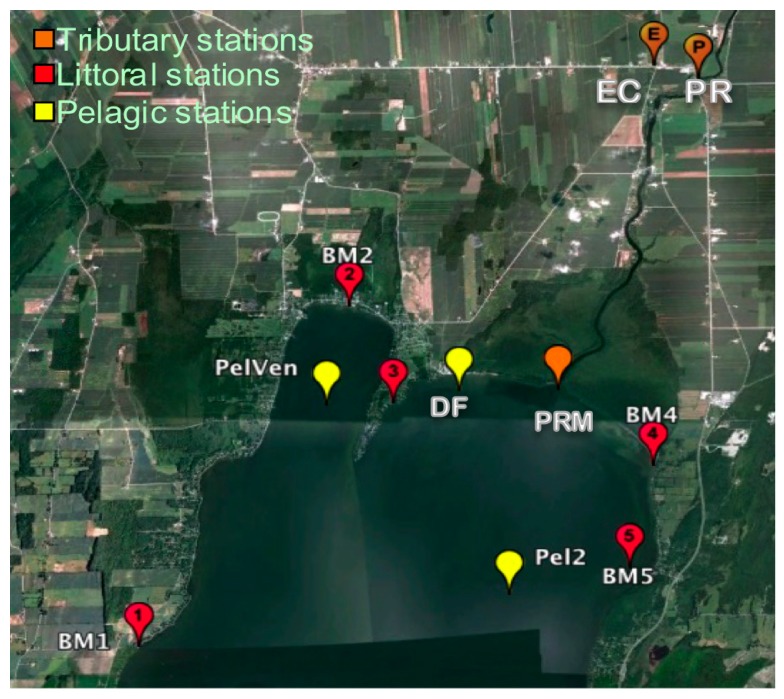
Sampling stations in Missisquoi Bay and its’ tributaries. Google Earth v. 7.0.1.8250. Missisquoi Bay, Lake Champlain. 45°02'44.86''N, 73°07'57.60''W, elevation 29 m. Eye altitude 29.50 km. Cnes/Spot Image 2012, DigitalGlobe 2012, GeoEye 2012. Available from: [72].

The samples for this study were collected from 11 stations in the Missisquoi Bay/Pike River area during 2009. There were five littoral (BM1, BM2, BM3, BM4 and BM5) and three pelagic stations (Pel 2 in Philipsburg, PelVen an DF) in Missisquoi Bay, one station located in the Pike River mouth (PRM) and two stations located in tributaries (Pike River (PR) and Ewing Creek (EC)) (Figure 2). When conditions allowed, the stations were sampled from April to December, except for the samples collected from the stations BM1 to BM4 and PelVen, which comprised part of an epidemiological study that was performed from the end of June to the end of August.

### 2.2. Sample Collection

For DNA analyses, the spring and fall samples from the shallow sampling sites BM5, Pike River and Ewing Creek were collected with a sterile glass bottle that was immersed beneath the surface of the water. For the pelagic stations, the photic zone was collected using a peristaltic pump. During the summer months, samples were collected for our study as well as an epidemiological study [5]. All of the littoral and pelagic stations were therefore sampled as follows: surface water was collected with an acrylic tube 93 cm long by 10 cm in diameter that was immersed horizontally beneath the surface of the water. Three sub samples were combined to form a composite sample.

For *E. coli* analyses, all samples were collected directly with a sterile bottle immersed beneath the surface of the water during the spring and the fall. In the summer, all of the littoral stations were sampled as follows: two samples were taken at the surface with a sterile bottle along two axis lines generally separated by about 10 meters. The two samples were combined in the lab to form a composite sample. The water samples were stored at 4 °C until further laboratory analysis, which took place within 48 h of sample collection.

For DNA analysis (*n* = 161), samples from both pelagic stations and the littoral station BM5 were collected on a weekly basis from April to June (the sampling of the pelagic stations started in May), every 3 to 5 days from July to August and weekly from September to November 10. The littoral stations BM1-BM4 were collected on a weekly basis from the third week of June to the end of August. DF was sampled monthly from May to November. Pike River Mouth was sampled monthly from May to June, biweekly during July and August and monthly in the fall. EC was sampled on a weekly basis during April, on a biweekly basis from May to July and once in August. PR was sampled on a weekly basis during April, on a biweekly basis from May to September and monthly in November and December. Due to inaccessibility, only stations BM5 and PR were sampled in December.

### 2.3. Cyanobacterial Abundance, E. coli Counts and Physico-Chemical Parameters

Phytoplankton subsamples (20 mL) were preserved with Lugol’s iodine. The identification and enumeration of cyanobacterial species were performed using an inverted microscope by the Utermöhl method [73].

For *E. coli* analyses, the water was filtered in duplicates within 24 h, on 0.45 µm cellulose ester membranes (Millipore Corporation, Bedford, MA, USA). The filters were processed in duplicates, on MI-Agar plates and incubated as recommended by the protocol MA.700-ECCtmi 1.0 from the “Centre d’expertise en analyses environnementales du Québec (CEAEQ)”. Fluorescent and blue colonies, equal to 0.5 mm or larger, were counted after a 24 h incubation period at 35 ± 0.5 °C [74].

Nutrient analyses were conducted at Université du Québec à Montréal (UQAM) in the laboratory of the Groupe de recherche interuniversitaire en limnologie et en environment aquatique (GRIL). Duplicate samples were taken for measuring nutrient concentrations. Samples for dissolved/bioavailable nutrients were taken from the filtrate of the GF/F 0.7 μm glass-fiber filter (Whatman, Inc., Florham Park, NJ, USA) used to filter water samples for intracellular toxins. Total and dissolved phosphorus concentrations were evaluated by the molybdenum-blue method after persulfate digestion and autoclaving. The digested samples were analyzed by spectrophotometry at 890 nm [75]. Total and dissolved nitrogen concentrations were measured as nitrates after alkaline persulfate digestion method coupled with a cadmium reactor [76]. Colorimetric analyses were carried out on an Alpkem-Flow Solution IV 0-1-Analytical RA autoanalyzer as described by the manufacturer (O-I-Analytical, College Station, TX, USA).

Water temperature was determined in the 4 m deep Pel2 station using HOBO Pro v2 data loggers, suspended at 0.02, 0.25, 1, 3 and 3.4 m. Temperature was recorded every minute, from 20 May to 28 October 2009. Because the lake was not thermally stratified, daily water column temperature was calculated as the average of all measurements (*n* = 7200 per day). Data from other stations and earlier dates were obtained with a submersible YSI 6600 v2 water quality multi-probe (YSI, Yellow Springs, OH, USA). Air temperature was recorded on site with a Kestrel 3500 weather meter (Nielsen-Kellerman, Bothwyn, PA, USA). Daily precipitation data for Philipsburg, QC (45°02'00.000"N, 73°05'00.000"W) was obtained from Environment Canada [77]. The data, recorded in mm, were summed for the preceding 7 days up to, but excluding, the day of sampling; it is referred to as 7-day precipitation in further analyses.

### 2.4. Microcystin Concentration Analyses

Microcystin analyses were evaluated by filtering 20 to 500 mL of water (depending on the density of the planktonic biomass) through a Whatman GF/F 0.7 μm glass-fiber filter (Whatman). Filtrand and filtrate were used to detect intracellular (particulate) and extracellular (dissolved) toxins respectively.

For intracellular toxins, a quarter of the filter was suspended in 1 mL of distilled water and sonicated on ice at full power for 1 min followed by centrifugation at 13,000× *g* for 10 min. Following centrifugation, the supernatant was collected for measurement. The extracellular toxin was measured in the GF/F 0.7 μm filtrate. The concentrations of microcystin were determined using the Microcystin/Nodularin (ADDA) ELISA kit, according to manufacturer’s instructions (Abraxis LLC, Warminster, PA, USA).

To avoid interferences present in the lake water the toxin extracts often had to be diluted 20 and 40 times for intracellular and extracellular toxins, respectively. If necessary, extracts were diluted to 50 times to enter the linear region of the standard curve estimated by nonlinear regression, based on a logistic model with four parameters. The detection limit was set at three times the standard deviation of the blank. Values below this limit were considered to be not distinguishable from zero.

### 2.5. Data and Statistical Analyses

The relationship between nutrients or *E. coli* counts with station type and rainfall, was analyzed based on the station type. A rank was assigned to each station type depending on its degree of contact with the shore: pelagic stations PelVen, Pel2, and DF, being furthest from the shore, were given a rank of 1, littoral stations BM1 to BM5 were given a rank of 2 and the tributary stations PR and EC were given a rank of 3. The PRM station was excluded from this analysis because it was the only station sampled at the delta of PR, and it did not fit in the rank criteria. In order to analyze the trend between station contact with the shore and the concentration of nutrients or *E. coli,* Cochran–Mantel–Haenszel (CSMH) statistics were performed and further verified with Spearman’s Rank correlations (*rs*). For CSMH, the continuous quantitative data for nutrient concentrations and the categorical data for station type contact with the shore were used, controlling for three rain levels (1 = 0–20 mm, 2 = 21–40 mm, 3 = 41–60 mm). For rs, the continuous quantitative data for nutrient concentrations and the categorical data for station type contact with the shore (1–3) were used. In order to analyze whether there was a relationship with nutrient concentrations/*E. coli* counts and rainfall, Spearman’s Rank correlations were performed using the continuous quantitative data for nutrient concentrations and rainfall (rain d-7) at each of the station types (*i.e.*, tributary, pelagic and littoral). A nonparametric analysis of variance, the Kruskal-Wallis test, was employed to evaluate whether the average annual nutrient concentrations or *E. coli* counts differed between station types. The statistical analyses were performed using SAS (v.9.3, SAS Institute Inc., Cary, NC, USA). Nonparametric scores were specified for all tests. Outputs with a *p* value < 0.05 were considered significant and their specific null hypothesis were rejected. Spearman Rank Correlations (*rs*) were calculated between the variables of interest employing R (v.2.15.0, The R foundation for Statistical Computing, Vienna, Austria). Two-tailed *p* < 0.05 was considered significant.

The analysis of major cyanobacterial taxa found over time and space and of the dynamics of *Microcystis*, the *mcyD* gene, and microcystin within the cyanobacterial community profile in relation to environmental factors during 2009, was done on the littoral, pelagic and Pike River Mouth stations of Missisquoi Bay, excluding the data from the tributary stations. Since water temperature (Tw) values did not differ significantly among the sampling stations, their daily average value was used for all the stations in the correlation analyses.

### 2.6. DNA Sample Preparation, Extraction and Quantification

Water samples were filtered (130–250 mL, depending on the amount of suspended solids and/or biomass) onto 0.2-μm hydrophilic polyethersulfone membranes (Millipore, Etobicoke, ON, Canada). The filters were kept frozen at −80 °C until processed. A chemical and enzymatic lysis method followed by hot phenol extractions was used to extract DNA [18]. For each sample, the DNA pellet was resuspended in 250 μL of TE (Tris-Cl, 10 mM; EDTA, 1 mM; pH 8). DNA quantification was performed using the PicoGreen^®^ dsDNA quantitation assay (Invitrogen, Burlington, ON, Canada) and a Safire microplate detection system (Tecan, Männedorf, Switzerland).

### 2.7. Quantitative PCR of Microcystis 16S rRNA and mcyD Genes

Primers that targeted a specific region of the *Microcystis* 16S rRNA gene were designed for quantitative PCR analyses [78]. Sequence alignments were performed with all of the 169 *Microcystis* sequences available in the NCBI database. Primers were designed using the NCBI Primer-BLAST program [79]. Primer specificity was validated using Primrose and OligoCheck programs [77]. Neither of the primers form self 3' dimers, hairpins or self-duplex. The primer UCyaF3 5'-AATACGGGGGAGGCAAGCGTTATC-3' and UCyaR3 5'-ACGCTTTCGCCACCGATGTTCTT-3' generated a 203 bp amplicon that was cloned to generate a standard curve for absolute quantification. The PCR reaction was performed in a 50 μL volume containing 0.5 μM of each primer, 3 units of *Taq* DNA polymerase (GE Healthcare, Baie d’Urfe, QC, Canada), 5 μL of 10X *Taq* polymerase buffer (100 mM Tris-Cl, pH 9.0, 500 mM KCl, 15 mM MgCl_2_), 1 mM MgCl_2_, 0.2 mM of each deoxynucleoside triphosphate and 10 ng of *M. aeruginosa* genomic DNA from a pure culture isolated from Lake Champlain. The PCR cycling conditions were as follows: prior to the addition of *Taq* DNA polymerase, the samples were denatured for 5 min at 96 °C. This was followed by 24 cycles of denaturing at 94 °C for 1 min, annealing at 60 °C for 1 min and extension at 72 °C for 1 min. The final extension was at 72 °C for 10 min. The PCR product was purified using the GFX ™ PCR DNA and Gel Band Purification Kit (GE Healthcare) and quantified by SYBR^®^ Safe staining (Qiagen, Mississauga, ON, Canada) and spot densitometry using a ChemiImager (Alpha Innotech Corporation, San Leandro, CA, USA). Ligation, transformation, and screening of transformants was performed as previously described [18]. Standards were made from tenfold serial dilutions of linearized plasmid DNA containing the UcyaF3/R3 fragment of the *Microcystis* 16S rRNA gene. The qPCR cycling conditions involved an initial preheating at 95 °C s for 15 min, followed by 45 cycles of 95 °C for 10 s, 60 °C for 15 s and 72 °C for 20 s. Quantitative PCR was performed on three fivefold dilutions of each DNA sample. The concentration of the 10° dilutions was verified by fluorescence using the PicoGreen^®^ dsDNA quantitation assay (Invitrogen). The limit of detection of the *Microcystis* 16S rRNA gene qPCR, was determined with the LOD tool from GenEx Pro software (version 4.4.2; MultiD Analyses AB, Göteborg, Sweden) as described by [18]. At a cutoff value of 33 cycles and 95% confidence level, the LOD was determined to be 31.15. The *Microcystis* qPCR data was adjusted based on the percentage of *Microcystis* sequences that were generated with the Ion Torrent sequencer using the UcyaF3/R3 primers (see section below). The resulting data were presented as *Microcystis* 16S rRNA copies/mL.

The dynamics of microcystin-producing cyanobacteria was evaluated with Q-PCR using the *mcyD* (*mcyD(KS)*) primers and conditions described in [18], with the exception that the annealing temperature was at 54 °C instead of 58 °C. The serial dilutions of plasmid DNA were also performed with the filtrate of 0.22 um lake water instead of deionized water to generate the standard curve. The PCR efficiency and the correlation coefficient of the standard curve were 1.00 and 0.99, respectively.

### 2.8. Cyanobacterial and Microcystis 16S rRNA Gene Amplification for High-Throughput Sequencing

PCR amplification was also performed with primers compatible with the Ion Torrent sequencing platform that target the 16S rRNA gene of cyanobacteria. Primers were designed based on the alignment of at least one representative sequence of the 139 cyanobacterial genera that were available in the NCBI database as of February 2012 [78]. Primers were designed with the Primer-BLAST program from NCBI and validated with Primrose and OligoCheck programs [80] and the MacVector software package version 10.6 (Accelrys, Cary, NC, USA). A pairwise similarity matrix was performed using MacVector to identify the cutoff values to properly differentiate the sequences between cyanobacterial orders. A total of 31 DNA samples were sequenced with the primer set UcyaF4/R6 [78]. The primer sequences are as follows: UcyaF4: 5'-GCAAGCGTTATCCGGAAT-3' and UcyaR6: 5'-TCTACGCATTTCACCGCT-3'. A pairwise similarity matrix analysis was also performed to identify the cutoff values to properly differentiate *Microcystis* up to the genus level with the UcyaF3/R3 primers. The UcyaF4/R6 and UcyaF3/R3 primer sets were supplemented with adapters as well as a unique (10 nt) multiplex identifier (MID) tag as described by [81]. The PCR reactions were performed as described previously with the following modifications: the reactions were prepared in 30 μL volumes and contained 0.75 U of *Taq* DNA polymerase (GE Healthcare), and 20 ng of sample DNA. PCR cycling conditions involved an initial denaturation at 95 °C for 5 min, followed by 25 cycles of 95 °C for 30 s, 53 °C for 30 s (60 °C for UcyaF3/R3 primers), 72 °C for 45 s and ending with a final extension step at 72 °C for 10 min. The 161 bp (cyanobacteria) and 203 bp (*Microcystis*) amplicons were purified from agarose gels using a QIAquick Gel Extraction kit (Qiagen), quantified using PicoGreen^®^ dsDNA quantitation assay (Invitrogen), diluted to a concentration of 5 × 10^8^ molecules and pooled in an equimolar ratio resulting in one pool. Sequencing was conducted on an Ion Torrent Personal Genome Machine™ using the Ion 314™ chip following the manufacturer’s protocols as described in [81].

The *Microcystis* cell abundance was estimated based on the *Microcystis* F3/R3 qPCR output. In this study, we assumed that *Microcystis* has 2 rRNA operons per genome [26,82]. The *Microcystis* 16S rRNA copies/mL values were combined with the percentage of *Microcystis* sequences obtained with the UcyaF4/R6 primers to determine the relative number of total cyanobacteria 16S rRNA gene copies/mL. The results were presented as estimated total cyanobacterial 16S rRNA copies/mL in further analyses.

### 2.9. Bioinformatic Analyses

The sequences were analyzed using the RDP pyrosequencing pipeline (http://pyro.cme.msu.edu/) [83]. The sequences were binned according to their MID tags (only accepting perfect matches). The MID tags and the primer sequences were trimmed. For cyanobacterial 16S rRNA gene amplicons, all sequences that had unidentified bases (Ns), an average expected quality score lower than 17, or were shorter than 150 bp, were removed from further analysis. The remaining sequences from the 57 distinct datasets were then submitted to the RDP Classifier tool, using a 0.5 bootstrap cutoff, in order to identify cyanobacterial sequences. The FASTA Sequence Selection tool from RDP was used to remove from further analysis any sequence that was not assigned to the phylum *cyanobacteria* in each dataset. The remaining sequences from each dataset were aligned, using the program BLASTN, against a cyanobacteria/chloroplast database (total of 3338 sequences of which 2852 were cyanobacterial sequences) downloaded from the RDP database as of February 2012. The 100 best hits from the BLASTN search were kept for each read in every dataset. The BLAST files were imported into MEGAN v4.70.4 [84] to assign the reads of each dataset to appropriate taxa in the NCBI taxonomy using the lowest common ancestor algorithm (LCA) with default parameters except: maximum number of matches per read, 100; Minimum Support, 1; Minimum Score, 44 (this score threshold removed hits with an E value higher than 10^−5^); Top Percentage, 0.5 (this parameter allowed to only analyze matches with the best score).

## 3. Results

### 3.1. Physico-Chemical Parameters in Missisquoi Bay and Its Tributaries

**Figure A1:**
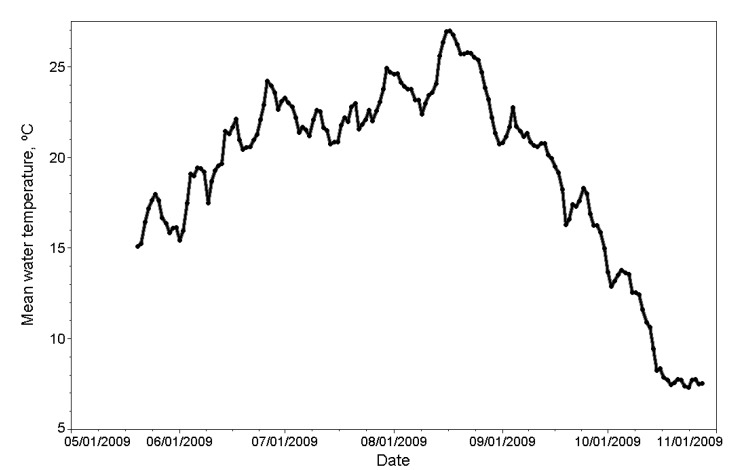
Temperature profile in the water column of the pelagic Pel 2 station from mid-May until the end of October. The thermistor was installed from the surface to the bottom, at depths 0.02, 0.25, 1, 3 and 3.4 m.

Temperature profiles at different depths in the pel2 station are presented in Figure A1 of the Appendix. Spring was characterized by a gradual increase of temperature interspaced with 4–5 °C drops in temperature. In the third week of June, the water temperature was already 24 °C. The highest water temperature was 27 °C and was recorded in mid-August.

**Figure 3:**
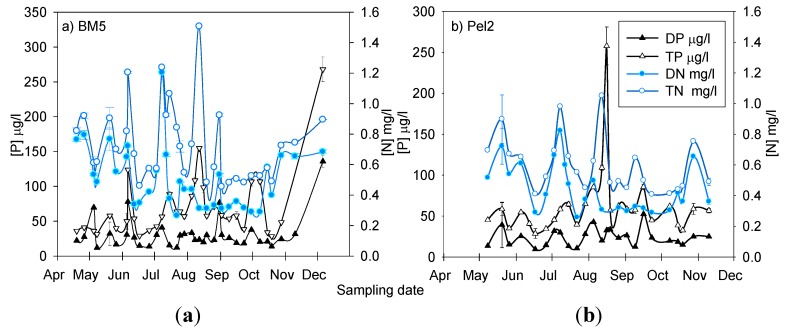
Nutrient concentrations over time in the Philipsburg areas (**a**) littoral station BM5 and (**b**) pelagic station Pel2.

**Figure A2:**
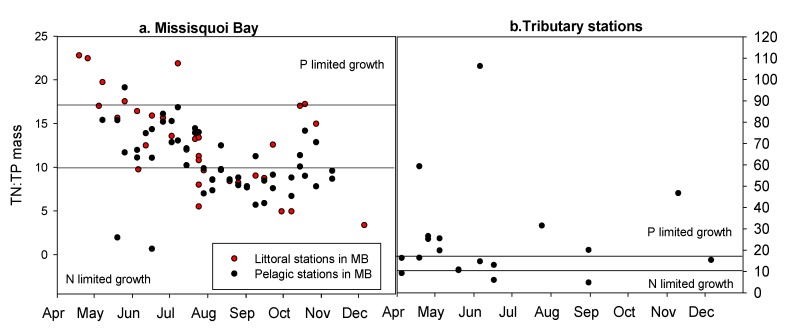
TN:TP ratio over time in Missisquoi Bay (**a**) and tributary stations (**b**). The horizontal bars represent the thresholds for N and P limited growth in phytoplankton as determined by Sakamoto [44]: N-limited growth TN:TP < 10:1 (mass); P-limited growth TN:TP > 17:1 (mass). At intermediate TN:TP ratios either nutrient could be limiting growth.

The nutrient concentrations in the littoral BM5 and pelagic areas of Philipsburg (Pel2) are presented in Figure 3. In general, eutrophic conditions were predominant in the bay throughout 2009, with an average TP concentration of 70.03 μg/L (Table 1). These concentrations far exceeded the TP annual mean concentrations recommended for this lake (25 μg/L) [54]. The mean dissolved phosphorus (DP) concentration observed in MB was 26.68 μg/L. The average TN and dissolved nitrogen (DN) concentrations were 0.64 mg/L and 0.45 mg/L, respectively. The nutrient concentrations for the tributary stations were higher than those observed in the bay. The average TP and DP were 157.73 μg/L and 61 μg/L, respectively. The average TN and DN concentrations were 2.5 mg/L and 2.4 mg/L, respectively. The TN:TP ratios observed during the sampling campaign are presented in Figure A2 of the Appendix. From May to the end of July, most samples had a TN:TP (mass) ratio between 10 and 17. In August and for the remaining part of the growing season, most of the TN:TP ratios were <10:1 (mass) indicative of a N limiting growth scenario.

**Table 1:** Average nutrient concentrations in the tributaries, littoral and pelagic stations of the Missisquoi Bay/Pike River area during the 2009 sampling campaign. DP (dissolved phosphorus); TP (total phosphorus); DN (dissolved nitrogen); TN (total nitrogen). The letters ^a,b,c^ identify which concentrations were significantly different between station types as determined by pairwise Kruskal-Wallis tests.

Parameter	Pelagic	Littoral	Tributaries
**DP (μg/L)**	23.12 ^a^	30.24 ^a^	61.00 ^b^
**TP (μg/L)**	69.21 ^a^	70.84 ^a,b^	157.73 ^b^
**DN (mg/L)**	0.39 ^a^	0.50 ^b^	2.40 ^c^
**TN (mg/L)**	0.53 ^a^	0.74 ^b^	2.50 ^c^

### 3.2. Spatial Analysis of Phosphorus and Nitrogen Concentrations and Their Relationship with Rainfall

**Figure 4:**
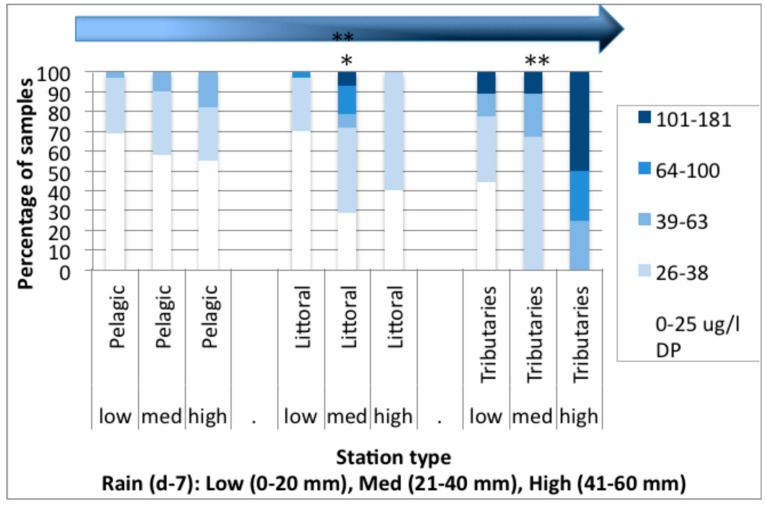
Dissolved phosphorus and its relationship with rainfall and station type. The arrows represent the positive trend observed between DP and station contact with the shore. Asterisks denote whether a significant correlation was found between rain and DP at each station type. (*) *p* < 0.05; (**) *p* < 0.01.

In order to verify whether nutrient input could be originating from external sources, such as surface runoff, nutrient concentrations were analyzed in relation to contact with the shore and rainfall. A rank was therefore assigned to each station type depending on the degree of contact with the shore. All the nutrient concentrations tended to significantly increase with increased contact with the shore. Strong and significant correlations were found between the concentrations of total and dissolved nitrogen and contact of the stations with the shore (Table 2). There was no significant correlation between rainfall and DN or TN in any of the station types (data not shown). The concentration of DP tended to significantly increase as contact with the shore increased; this correlation was stronger than the one observed with TP (Table 2). The correlation between rain and TP was strong and significant but only in the tributary stations (*rs* = 0.65, *n* = 23, *p* < 0.009). The correlation between rain and DP was strong and significant in the tributary stations (*rs* = 0.55, *n* = 22, *p* < 0.008) as well as in the littoral stations (*rs* = 0.37, *n* = 45, *p* < 0.01). The results for dissolved phosphorus are presented in Figure 4.

**Table 2:** Relationship between increasing contact of the station type to the shore and nutrient concentrations as determined by Cochran–Mantel–Haenszel (CMH) and Spearman Rank correlations. DP (dissolved phosphorus); TP (total phosphorus); DN (dissolved nitrogen); TN (total nitrogen).

Parameter	Cochran–Mantel–Haenszel (CSMH Correlation/p)	Spearman Rank Correlations (Rho Estimates/n/p)
**DP**	18.72 (*p* < 0.0001)	0.40/126 (*p* < 0.01)
**TP**	4.37 (*p* = 0.0366)	0.21/121 (*p* < 0.05)
**DN**	48.46 (*p* < 0.0001)	0.64/119 (*p* < 0.01)
**TN**	55.63 (*p* < 0.0001)	0.72/111 (*p* < 0.01)

### 3.3. Nutrient Input in Relation to E. coli Abundance and Cyanobacterial Blooms in Missisquoi Bay and Pike River

**Figure 5:**
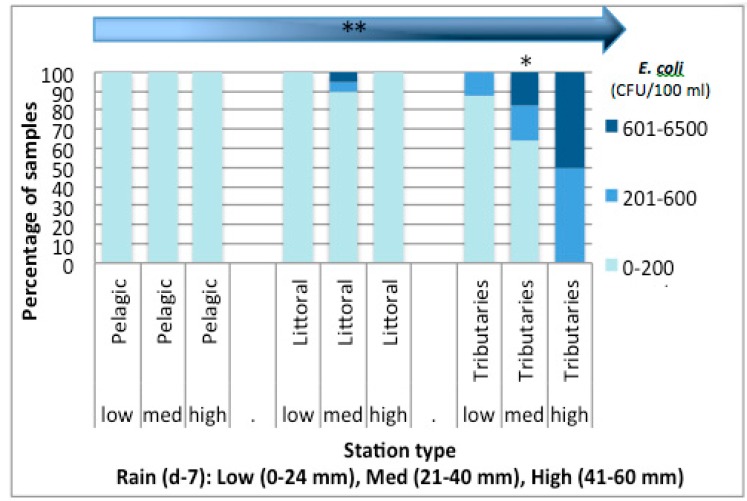
*E. coli* counts and their relationship with rainfall and station type in Missisquoi Bay during the 2009 sampling campaign. The arrow represents the positive trend observed between *E. coli* counts and station contact with the shore. Asterisks on top of the bars denote whether a significant correlation was found between rainfall and *E. coli* counts (colony forming units (CFU)) at each station type (*) *p* < 0.05; (**) *p* < 0.01.

To evaluate whether nitrogen and phosphorus external loads contribute significantly to the nutrient concentrations observed in the bay, coliform and *E. coli* counts were used as an indicator of nutrient input from external sources, such as nutrient surface runoff or sewage overflows. The highest concentrations of coliforms and *E. coli* were detected in the spring and in the fall (Table 3). The sampling season average *E. coli* counts were 3.65 colony forming units (CFU)/100 mL in the pelagic stations; 92.82 CFU/100 mL in the littoral stations; and 528.57 CFU/100 mL in the tributary stations (data not shown). Positive correlations were observed between *E. coli* counts and the dissolved and total nutrients (Table 4). The averaged *E. coli* counts for each type of station was significantly different between the littoral and tributary stations (Kruskal Wallis test Chi sq = 16.99, *p* < 0.0001) and between the pelagic and tributary stations (Kruskal Wallis test Chi sq = 23.35, *p* < 0.0001). The *E. coli* counts tended to increase as the station contact with the shore increased (CSMH correlation = 23.45, *p* < 0.0001; *rs* = 0.57, *n* = 79, *p* < 0.0001) (Figure 5). The correlation between rainfall and *E. coli* counts was only significant in the tributary stations (*rs* = 0.48, *n* = 34, *p* < 0.03). *E. coli* counts also correlated significantly (*rs* = 0.41, *n* = 34, *p* = 0.0172) with cyanobacterial cell abundance (data not shown).

**Table 3:** *E. coli* and coliform counts in Missisquoi Bay during the 2009 sampling campaign. TNC; (Too Numerous to Count). NSW; (No Sampling due to Weather). Samples that corresponded to *E. coli* counts higher than 200 colony forming units [CFU]/100 mL are highlighted in orange.

DATE	*E. coli*; Coliform Counts at Station:										
	Lit BM1	Lit BM2	Lit BM3	Lit BM4	Lit BM5	Pel Ven	Pel 2	EC	PR	PRM	DF
19 April 2009					0;0		25;85	70;260	25;85		
26 April 2009					0;0			20;60	70;620		
5 May 2009					0;92			20;160	10;455		
8 May 2009					0;278		4;78				
20 May 2009					8;998		2;70	170;2020	185;2125	42;752	0;6
6 June 2009					24;928			540;5120	60;920		
17 June 2009					0;64		0;TNC	820;TNC	470;TNC	655;TNC	12;736
30 June 2009					0;74						
19 July 2009	425;2725	2410;TNC	90;3490	10;TNC	5;1005	0;TNC	NSW				
25 July 2009	5;TNC	50;TNC	5;TNC	0;0	0;TNC	0;545	0;1750		85;TNC	10;TNC	0;2400
1 August 2009	35;TNC	125;1850	0;90	10;TNC	0;665	NSW	NSW				
8 August 2009	0;950	35;1385	0;20	0;815	5;515	0;230	0;0		55;2595		0;205
16 August 2009	0;2400	120;1120	0;2750	0;1900	0;1000	0;110	0;155				
22 August 2009					0;140	0;210	15;415	5;1555	0;210		
31 August 2009					10;550			TNC;TNC	1420;TNC		
23 September 2009					5;650		25;530		240;810		10;1395
10 November 2009					0;500	0;140	0;305		5;950	0;25	5;60
6 December 2009					150;TNC				330;TNC		

**Table 4:** Relationship between *E. coli* counts and nutrient concentrations as determined by Spearman Rank correlation on the 2009 data from the Missisquoi Bay/Pike River area. The data is displayed in the following order: Spearman rho estimates (*rs*)/number of data points in the correlation tested (n)/*p*-value. DP (dissolved phosphorus); TP (total phosphorus); DN (dissolved nitrogen); and TN (total nitrogen). For the correlations, the quantitative data was used for all parameters.

	TP	DN	TN	*E. coli*
**DP**	0.48/122 (*p* < 0.01)	0.50/117 (*p* < 0.01)	0.40/111 (*p* < 0.01)	0.51/61 (*p* < 0.01)
**TP**		0.23/109 (*p* < 0.01)	0.44/106 (*p* < 0.01)	0.36/56 (*p* < 0.01)
**DN**			0.82/114 (*p* < 0.01)	0.65/52 (*p* < 0.01)
**TN**				0.63/46 (*p* < 0.01)

### 3.4. Spatio-Temporal Characterization of Major Cyanobacterial Taxa and Their Association with Environmental Parameters in Missisquoi Bay/Pike River during 2009

**Figure 6:**
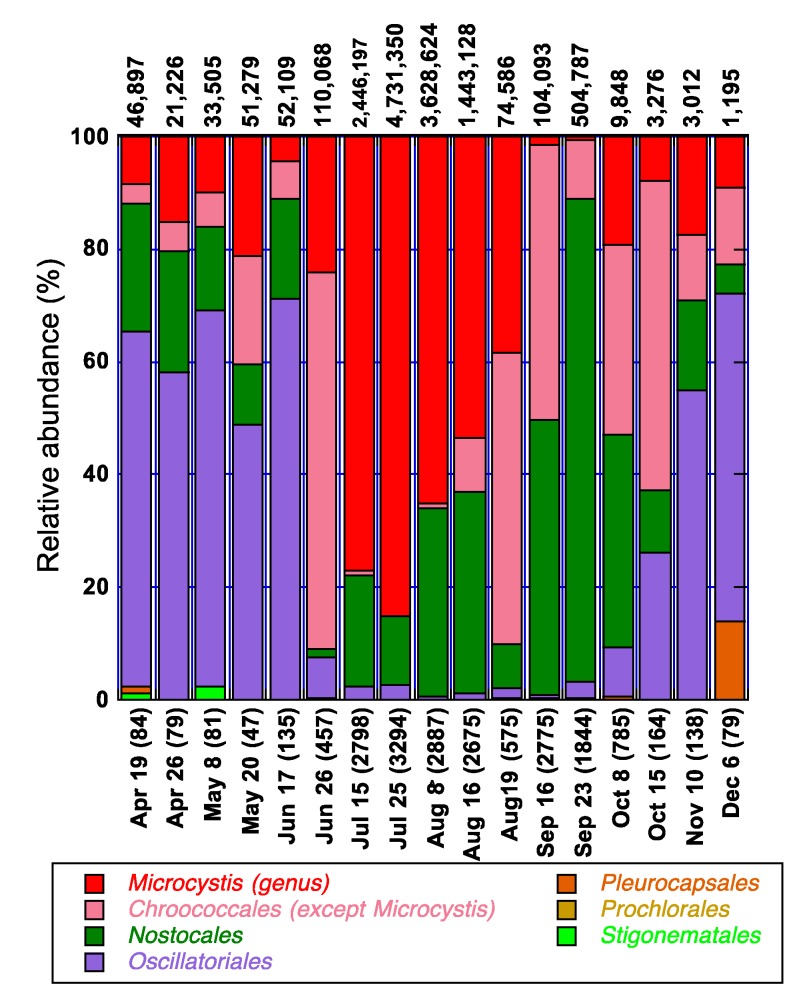
Temporal variation of *Microcystis* in relation to cyanobacterial orders in 2009 in the littoral station (BM5). The number of total cyanobacterial sequences generated at the order level is indicated in parenthesis (x-axis). The numbers on top of the bars represent the estimated total cyanobacterial 16S rRNA gene copies/mL in the sample.

**Figure 7:**
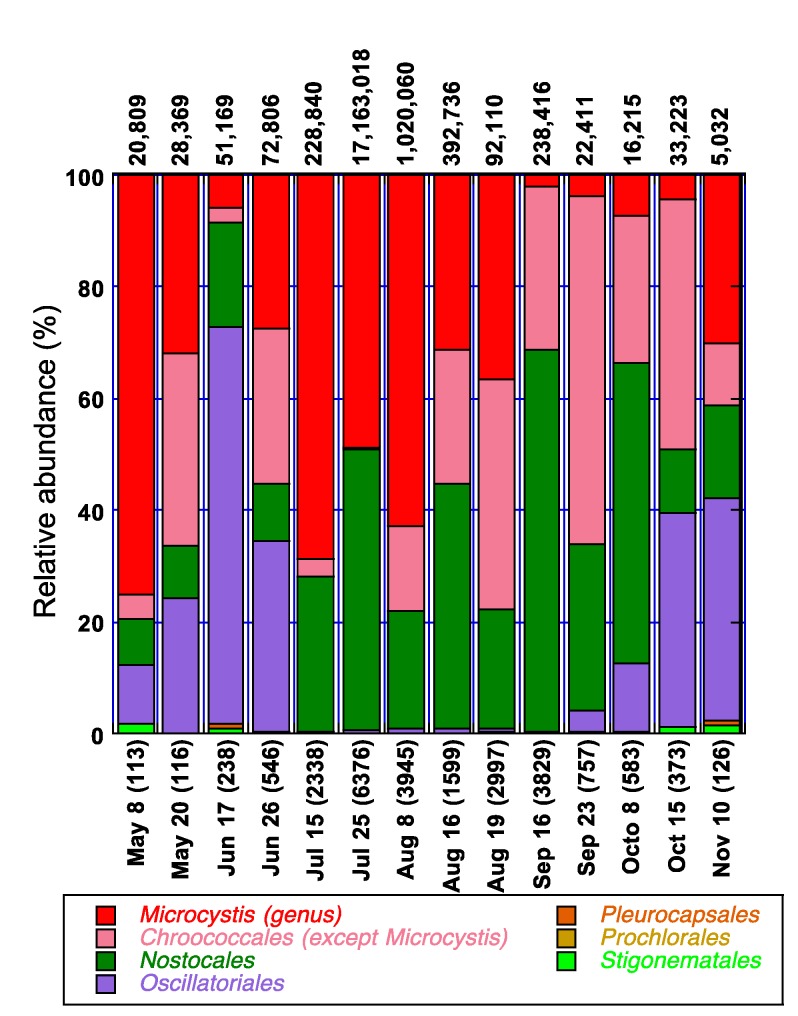
Temporal variation of *Microcystis* in relation to cyanobacterial orders in 2009 in the pelagic station (Pel 2). The number of total cyanobacterial sequences generated at the order level is indicated in parenthesis (x-axis). The numbers on top of the bars represent the estimated total cyanobacterial 16S rRNA gene copies/mL in the sample.

The summer of 2009 was associated with a severe and persistent cyanobacterial bloom. High-throughput amplicon sequencing of the 16S rRNA gene was performed on samples collected from the littoral (BM5) and pelagic stations (Pel2) of Philipsburg (Figure 6 and Figure 7). All the cyanobacterial orders, except for the *Gloeobacterales*, were observed in all of the stations at some point. The major orders were *Chroococcales*, *Nostocales* and *Oscillatoriales* in both stations*.* In the *Chroococcales* order, the predominant genera were *Microcystis* and *Synechococcus*. The predominant genera in the *Oscillatoriales and Nostocales* orders were *Leptolyngbya* and *Dolichospermum* [85], formerly known as *Anabaena* [86], respectively (data not shown)*.*

In the littoral station, the community appeared to be dominated by members of the *Chroococcales*, *Nostocales* and *Oscillatoriales* (Figure 6)*.* The genus *Microcystis* (*Chroococcales*), was present in most of the samples analyzed throughout the sampling campaign. It was already observed in the water column on April 19, with a relative abundance of 8%. The total phosphorus and nitrogen concentrations in the littoral BM5 of Philipsburg that day were 36 μg/L and 0.82 mg/L, respectively (data not shown). Ninety three and 61% of the N and P were in the dissolved/bioavailable form, respectively. At the beginning of May, *Microcystis* already constituted more than 70% of the cyanobacterial community in the pelagic station (Figure 7). However, its relative abundance in the littoral area was only 10%. On May 20, an increase in TP (from 35.95 to 57.8 μg/L) was accompanied by a relative decrease in the dominance of *Oscillatoriales* and an increase in *Chroococcales* (Figure 6). Late June was associated with a temperature increase to 24 °C (Figure A1 of the Appendix), a decrease in phosphorus concentration (Figure 3) and a shift of dominance in both stations towards the *Chroococcales*, where genera other than *Microcystis* prevailed (Figure 6 and Figure 7). Taxonomy analyses revealed that the population of the non-toxic species *Aphanothece clathrata brevis* in the water column was substantial in both the pelagic and littoral areas of Philipsburg (data not shown). The abundance of *Microcystis aeruginosa* in these areas constituted only 17% and 1.7% of the population (data not shown).

An important cyanobacterial bloom took place during July and August in both the littoral and pelagic areas of Philipsburg. The *Chroococcales* and the *Nostocales* dominated the water column during that episode. On July 25, the numbers of cyanobacterial 16S rRNA gene copies in the littoral and pelagic stations were more than 4.7 × 10^6^ and 17.2 × 10^6^ copies/mL, respectively (Figure 6 and Figure 7). *Microcystis* constituted 85% of the bacterial community in the littoral station. The water temperature that day was 21 °C (Figure A1 of the Appendix), TP = 64.00 μg/L, and TN = 0.72 mg/L (Figure 3). The relative abundance of *Microcystis* was 50% in the pelagic area. Members of the *Nostocales* order represented the other half of the population. Taxonomy analyses revealed that the *Nostocales* species observed during that time included *D. spiroides* [87], *Aphanizomenon flos aquae* and *Aphanizomenon flexuosum* (data not shown). While there were no significant differences between the annual averages of the environmental parameters analyzed in the two sampling stations (data not shown), the TN concentrations were significantly higher at BM5 (0.72 mg/L). In September, the *Microcystis* population subsided in both stations, where the concentrations of TN were below 0.52 mg/L and the TN:TP mass was below 9:1. A smaller bloom event was observed in both the littoral and pelagic areas of Philipsburg in mid-September. *Nostocales* and *Chroococcales* other than *Microcystis*, mainly dominated the cyanobacterial community. Taxonomy analyses demonstrated that the predominant *Nostocales* species in both stations was *D. spiroides* ([85] ex [87]), which constituted 53% and 71% of the cyanobacterial population in the pelagic and littoral areas, respectively (data not shown). The *Nostocales* dominance in Pel2, corresponded to the following conditions: water temperature = 17–19 °C (Figure A1 of the Appendix); DN = 0.32 mg/L; TN = 0.50 mg/L; TP = 85.56 μg/L and the TN:TP mass = 6:1. In BM5, *Nostocales* reached its dominance peak towards the end of September when the water temperature was 18 °C (Figure A1 of the Appendix); DN = 0.32 mg/L; TN = 0.48 mg/L; TP = 38.51 μg/L and the TN:TP mass was about 13:1.

As the temperature started to decrease in October, the relative abundances of the *Chrococcales* in both stations and of the *Nostocales* in the littoral decreased. *Oscillatoriales* abundance increased and this order dominated the cyanobacterial population. In November, the genus *Microcystis* however, showed a 25% increase in relative abundance in the pelagic area of Philipsburg which was consistent with an increase in nutrient concentrations as follows: TN = 0.49 mg/L; TP = 56.74 μg/L; TN:TP mass = 9:1.

**Figure 8:**
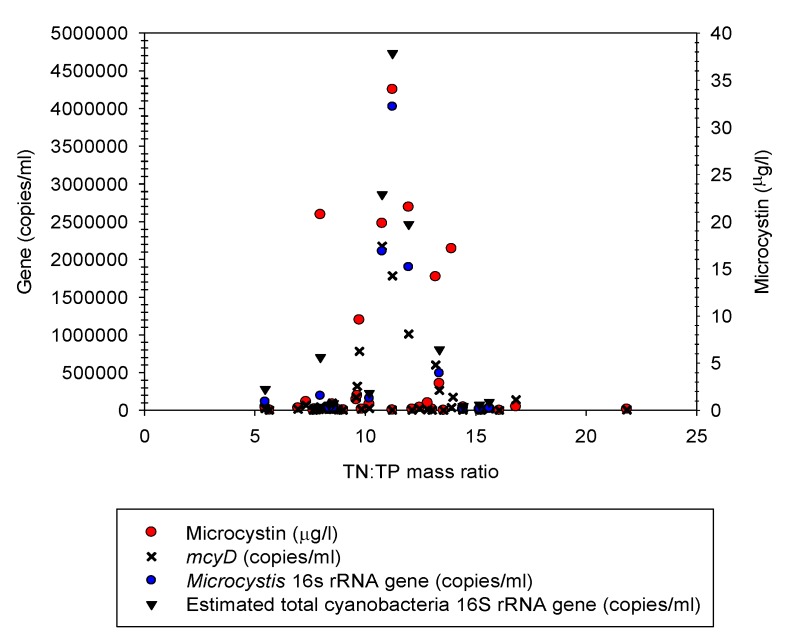
Relationship between the TN:TP mass ratio and intracellular microcystin concentrations, *mcyD*, total cyanobacteria and *Microcystis*16S rRNA gene copies during the 2009 sampling campaign in Missiquoi Bay. The graph represents samples that were collected at water temperatures ≥ 20 °C.

The TN:TP relationship between *Microcystis* relative abundance and the nutrient ratios was evaluated with all the samples that were collected in Missisquoi Bay/Pike River at water temperatures higher than 20 °C. For this analysis, it was assumed that water temperature of ≥20 °C did not limit the growth of *Microcystis* because concentrations of more than 200,000 16S rRNA gene copies/mL were observed at ≥20 °C. The selected samples had nutrient concentration ranges of 34.48 to 162.89 μg/L for TP and 0.48 to 1.15 mg/L for TN (data not shown). Interestingly, it was observed that regardless of the sampling date or station, the relative abundance of *Microcystis* increased as the TN:TP (mass) ratio approached 11:1 (Figure 8).

### 3.5. Microcystin Analyses

Of the 349 samples collected for intracellular toxin analysis, 65.9% were below the WHO [52] provisional drinking-water guideline value of 1.0 μg/L microcystin-LR (data not shown). Thirteen percent of the samples had concentrations between 1–4 μg/L, and between 4–20 μg/L. The toxin concentration profile in all of the stations during our 2009 sampling campaign was July > August > September. In mid-July, the microcystin concentration in the littoral area of Philipsburg was 34.8 μg/L in the subgroup of samples where water temperatures were ≥20 °C (Figure 8). Analyses of extracellular toxins (*n* = 308) revealed that 94.5% of the samples were below the drinking-water guideline and only 3 samples had extracellular toxin concentrations higher than 4 μg/L (data not shown).

### 3.6. Q-PCR Analyses of Microcystis 16S rRNA and mcyD Genes

In the littoral BM5 and pelagic (Pel2) stations of Philipsburg, the dynamics of the *Microcystis* 16S rRNA gene copies/mL closely followed the pattern of estimated total cyanobacteria 16S rRNA copies/mL, *mcyD* gene and microcystin, especially during July and August when the *Microcystis* bloom took place (Figure 8). The *Microcystis* 16S rRNA gene copies per mL significantly and positively correlated with both intracellular (*r* = 0.89, *n* = 33, *p* < 0.01) and extracellular microcystin concentrations (*r* = 0.54, *n* = 20, *p* = 0.05) as well as *mcyD* gene copies/mL (*r* = 0.96, *n* = 42, *p* < 0.01). Positive correlations were not observed for the other potential toxin producing genera that were identified, such as *Leptolyngbya* and *Dolichospermum* [85]. This suggests that *Microcystis* was the main microcystin producer in the bay.

All of the samples with *Microcytis* cell numbers higher than 100,000 cells/mL were concomitant with microcystin concentrations higher than 20 μg/L. Only one sample out of 100 had a microcystin concentration higher than 20 μg/L (20.7 μg/L) and less than 100,000 *mcyD* gene copies/mL (46,782 ± 1613 copies/mL). The microcystin content per *mcyD* gene copy (estimated toxic cell) ranged from 1.33 to 1455 fg/copy. The average microcystin content per *mcyD* copy (estimated toxic cell) was 61.28 fg/copy, while the median value was 18.40 fg of microcystin per *mcyD* gene copy.

## 4. Discussion

### 4.1. Nutrient Input in Relation to Rainfall, E. coli and Cyanobacterial Blooms

Surface runoff contributed substantially to the nutrient load in MB and Pike River during 2009. Total and dissolved phosphorus concentrations increased in the stations closest to the shore and a significant correlation was detected between rainfall and P concentrations in the tributary stations. The higher concentrations of nutrients observed in the littoral and tributary stations compared to the pelagic areas may have also originated from re-suspension of P from the sediments [22,41,88] a scenario associated with mixing events or strong winds in shallow aquatic systems.

The concentrations of N also increased in the stations closest to the shore. Significant correlations were not detected however, between N concentrations and rainfall near the tributary stations. This suggests that the flow pathway for nitrogen may be influenced by other parameters. For example, N has been shown to be leaching via mechanisms such as infiltration, water percolation and irrigation. Over the years, agricultural practitioners have increasingly installed subsurface tile drainage systems to lower seasonally high water table levels and consequently substantially increase crop yields in poorly drained soils. These changes have contributed to the increase of both nitrogen and phosphorus loads into tributaries. Enright and Madramootoo [89] demonstrated that subsurface flow through tile drains could account for 40% of the total P exported from fields located in the Missisquoi Bay/Pike River watershed. While phosphorus can be strongly adsorbed to soil particles, nitrates are highly soluble. Higher concentrations of N compared to P have been reported in tile drain effluents [90,91]. A study by Madramootoo and colleagues [58], performed in sandy and clay loam soils in the same watershed has revealed that 62 and 73% of the total nitrogen was being exported as nitrate via tile drains, respectively. Another study performed in fields with clay loam soils, have shown that subsurface flow pathways contributed up to 90% of the water and N through the soil profile and into the tile drains [92].

The application of manure on the fields during the late spring and the fall is a common practice in the Missisquoi Bay/Pike river watershed. Runoff of phosphorus and dissolved/bioavailable forms of N such as ammonia and nitrate from manure are often observed during these periods because the agricultural fields are bare or contain fewer plants. Interestingly, the highest concentrations of nutrients and high coliforms and *E. coli* counts were observed in the tributaries and in the Pike River mouth during these seasons. These results suggest that considerable inputs of P and N originated from surface and subsurface runoffs of manure and fertilizers. Michaud and colleagues [93] estimated that 65% of the phosphorus load in the Missisquoi Bay/Pike River watershed originates from farm fertilizers. A ton of fresh manure from most animals contains roughly 4 to 9 kg of nitrogen, 2 to 4 kg of phosphorus and 4 to 7 kg of potassium [94]. This type of runoff in addition to sediment and organic solids, can transport pathogens, such as *E. coli* to surface waters [95]. In this study, significant correlations were detected between *E. coli* and the concentrations of nutrients. The *E. coli* counts also significantly increased with stations closest to the shore and a correlation was observed between *E. coli* counts and rainfall in the tributary stations. It is important to note that coliforms and *E. coli* in the water column can also originate from other potential sources, such as runoff from defective septic systems, saturated drain fields as well as sewage overflows following intense rainfall. In 2009 alone, there were 155 overflow events from the wastewater treatment plant in Bedford: 137 associated with rain, 13 associated with snow melt and 5 associated with damage and/or repair work [96].

The bioavailability of ammonia can be extremely relevant to predict cyanobacterial blooms since this form of N has been shown to control all N-related biochemical functions in cyanobacteria, including toxin production [97]. The presence of ammonia in the water column can therefore provide cyanobacteria with a competitive advantage over the rest of the phytoplankton community. In this study, we witnessed an early *Microcystis* bloom at the beginning of May in the pelagic area of Philipsburg. A week prior to this sampling campaign, a 19 mm rainfall event gave rise to significant inputs of coliforms and bioavailable nutrients into Pike River and into the bay. Similarly, a 10% increase in *Microcystis* relative abundance was observed on May 20 in the BM5 station following a 26 mm rainfall event on May 16. Substantial concentrations of dissolved/bioavailable nutrients, *E. coli* and coliforms were observed in the tributaries. The amount of nutrients and coliforms in the BM5 littoral area of Philipsburg was also significant. These results suggest that *E. coli* counts is a good indicator of recent nutrient input from human and animal excreta as well as surface and subsurface runoffs.

### 4.2. Absolute Nutrient Concentrations, Nutrient Ratios, and the Dynamics of Microcystis

It has been suggested that the shift to cyanobacterial dominance within the phytoplankton community in the Vermont part of the bay, may have been the result of increasing water temperatures, increasing P loads from agriculture and urbanization and decreasing TN levels, resulting in a decrease in the TN:TP ratio [51]. Pearce and colleagues [98] showed that the low dissolved nitrogen to soluble reactive phosphorus and anoxic sediments contributed to the set of blooming conditions in the Vermont area of Missiquoi Bay during the summers of 2007 to 2009.

In this study, the relative abundance of *Microcystis* in the Quebec portion of Missisquoi Bay was significantly correlated with temperature, TP and TN. The genus *Microcystis* was generally dominant (relative abundance in samples was higher than 50%) at TP concentrations higher than 39 μg/L, TN levels equal to or higher than 0.52 mg/L and temperatures higher than 14 °C, independently.

The dynamics of *Microcystis* in Missisquoi Bay corresponded to a non-linear non-monotonic relationship with nutrient ratios. At water temperatures equal to or higher than 20 °C, the dominance of *Microcystis* tended to increase as the TN:TP (mass) ratio approached 11:1, irrespective of the sampling date or station. The occurrence of *Microcystis* blooms in the bay during subsequent years also corresponded to TN:TP ratios in this range (data not shown). For example, a TN:TP ratio of 11:1 was observed on July 12, 2010 in the pelagic area of Philipsburg. In the littoral station, the TN:TP ratio reached a value 11:1 on July 26 and remained in this range until October 3. During the spring of 2011, there was an historical flood event that led to unusually high TN:TP ratios in the bay (between 25:1 to 52:1). A TN:TP ratio approaching 11:1 was observed only in the littoral area of Philipsburg during our sampling campaigns of August 4 and October 2 (data not shown).

The importance of absolute nutrient concentrations and ratios on cyanobacterial dynamics has been previously and widely recognized [33,35,45,46,99,100,101,102,103]. Our observations are in agreement with the concept that stoichiometric requirements need to be fulfilled in order for algae to grow, since the biochemical function of each nutrient is unique and cannot be substituted [33]. Uni-algal laboratory experiments have demonstrated that optimum atomic TN:TP ratios are species-specific [101]. For example, an optimum TN:TP atomic ratio of 9:1 was identified for *Microcystis* sp. in their study. Similarly, [102] determined that a TN:TP atomic ratio of 11 was ideal for the growth of a *Microcystis aeruginosa* strain isolated from flooded rice paddies. Previous field studies have suggested that low nitrogen to phosphorus ratios favor cyanobacterial dominance in freshwater lakes [46] and that *Microcystis* tend to dominate the phytoplankton community at TN:TP (mass) ratios below 30:1 [27]. Finally, Kotak and colleagues [36] demonstrated that the growth of *Microcystis* strain PCC7820 was stimulated with a decreasing TN:TP ratio in the medium.

It has been suggested that in a phytoplanktonic assemblage the nutrient ratios determine the species composition and their relative biomass while the absolute nutrient concentrations determine the total biomass [103]. In line with this suggestion, *Microcystis* dominance and abundance as determined by the number of *Microcystis* 16S rRNA gene copies/mL was generally higher at nutrient ratios approaching 11:1. We suggest that the dynamics of *Microcystis* in MB during 2009 were determined by both absolute nutrient concentrations and their ratio, in such a way that once temperatures were favorable for *Microcystis* to thrive, the dominance of *Microcystis* depended on the stoichiometric relation between N and P while the absolute nutrient concentrations influenced the amount of biomass that could be supported.

### 4.3. Major Cyanobacterial Taxa and Their Distribution Dynamics

The major cyanobacterial taxa and potential microcystin producers were determined by 16S rRNA gene high-throughput sequencing using the Ion Torrent platform. To our knowledge, it is the first time that next-generation amplicon sequencing has been used to characterize cyanobacterial blooms.

Members of the *Nostocales* family tended to co-exist with *Microcystis* at similar TP concentrations during both the spring and summer. In early May and July, the TP and temperature conditions were favorable for either taxon to dominate, but the nitrogen concentrations were high (TN > 0.52 mg/L) and the nutrient (mass) ratios were close to 11:1. These conditions favored the dominance of *Microcystis* over *Nostocales* and the rest of the cyanobacterial community.

It is possible that the simple presence of microcystin in the water column affected the growth of *Nostocales* and contributed to the persistence of *Microcystis* during the summer. An allelopathic effect of microcystin on the growth of *Dolichospermum* [85] has been previously suggested based on observations from competition experiments between toxic *Microcystis* *aeruginosa* and *Anabaena* sp. PCC7120 [104]. However, the allelopathic effects of microcystin on cyanobacterial population dynamics remain highly controversial [104,105,106,107]. Further research is clearly needed in this area.

In this study, the *Nostocales* eventually peaked during September once the main *Microcystis* bloom had subsided to <5000 *Microcystis* 16S rRNA gene copies/mL. The decline of *Microcystis* was concomitant with a decrease in temperature and nitrogen concentrations, with TN:TP mass ratios of 6:1 and 9:1 for the pelagic and littoral stations, respectively, suggesting N-limitation. The shift in dominance can probably be attributed to the capabilities of *Nostocales*, such as *Dolichospermum* [85] and *Aphanizomenon* to fix nitrogen [108,109], as well as their ability to tolerate lower temperatures than *Microcystis* [110]. Consistent with our study, the nutrient enrichment experiments performed by Gobler and colleagues [22] showed that the decline of *Microcystis* could be attributed to N-limitation. Other studies have also suggested that nitrogen limitation can lead to a reduction in buoyancy and sinking of *Microcystis* cells [111,112], since nitrogen is necessary for the production of proteins that are required for gas vesicle formation [97].

### 4.4. Toxin and Q-PCR Analyses of 16S rRNA and mcyD Genes

In this study, total cyanobacterial cells, microcystin concentrations, as well as *Microcystis* 16S rRNA and *mcyD* gene copies/mL were mutually, positively and significantly correlated in MB. This is in agreement with previous correlation analyses done on Lake Erie samples [26]. In our study, the total cyanobacterial 16S rRNA gene copies/mL were highly correlated with intracellular microcystin concentrations. The intracellular and extracellular microcystin concentrations, as well as the *mcyD* gene copies/mL, were positively and significantly correlated with the genus *Microcystis* suggesting that the main microcystin producers in the system were members of this genus. Similarly, the multiplex quantitative PCR assay developed by Ngwa and colleagues revealed significant associations between *Microcystis* cell counts and *mcyA*, *mcyE* and *mcyG* gene copies/mL in Missisquoi Bay during the summer of 2009 [113]. The same authors demonstrated that microcystin concentrations correlated significantly with *mcyE* gene copies/mL as well as with the *Microcystis* biomass during the cyanobacterial bloom of 2011 [114]. In their study, *Microcystis* was identified as the main microcystin-producing species in the bay.

Several studies performed in freshwater lakes revealed that microcystin concentration dynamics were also associated with changes in the concentrations and ratios of N and P [36,115]. Analyses of 246 freshwater Canadian lakes from 2001 to 2011 revealed high microcystin concentrations only at low N:P ratios (<23:1 reported as by mass) [116]. A negative relation between microcystin concentrations and N:P ratios was also reported in several Alberta lakes [36] and Lake Erie [26].

### 4.5. The Impact of Climate Change on Cyanobacterial Blooms

Over the period of 1964 to 2009, the average surface temperatures in Lake Champlain increased by 1.6–3.8 °C during the month of August [51]. There is a general consensus that changes in precipitation patterns and rising global temperatures are expected to lead to increased nutrient loading from surface runoff, the dominance of cyanobacteria and the incidence of toxic blooms [28,34,38,42,117,118,119,120,121]. The high temperature optimum growth rates in cyanobacteria become a competitive advantage allowing them to outcompete other members of the phytoplankton community during warm periods [38]. Previous studies found that phosphorus and nitrogen concentrations [22,42,122], as well as temperature [27,42] are factors that have a significant and positive effect on the biomass of *Microcystis.* For example, *Microcystis* has been observed to grow optimally at temperatures equal to or higher than 25 °C [27,42,110,112,123,124,125,126]. During our 2009 sampling campaign, quantitative PCR analyses of the 16S rRNA gene revealed that *Microcystis* populations could reach the WHO guideline [52] for moderate probability of adverse health effects (100,000 cells/mL) when temperatures were higher than 20 °C.

There are other indirect effects of temperature that can result in cyanobacterial dominance. In shallow reservoirs, warming of the sediment-water interface can indirectly promote cyanobacterial dominance by accelerating P release from the sediments [51,127]. Substantial spikes of nutrients from the sediments can also be triggered by strong winds and the resulting wave action [41]. Extreme weather events associated with climate change are often concomitant with strong winds and are likely to contribute to the resuspension of nutrients, especially in shallow lakes, rivers ponds and reservoirs.

### 4.6. Transboundary Challenges

Lake Champlain is the sixth largest inland freshwater body in the United States. Its basin is a 21,150 km^2^ drainage area [128] that encompasses regions of Vermont (56%), New York (37%) and Quebec (7%) [49]. The Missisquoi Bay watershed is divided between Quebec (42%) and Vermont (58%). Despite sustained efforts and financial investments at the provincial, federal, state and international levels, a decrease in phosphorus concentrations in the shallow bays and in the lake has not been observed [51,58,129,130]. Improvements have been detected, however, in some of the tributaries [131,132].

In 2011, two historic flood events in the spring and summer contributed to an extraordinary spike in phosphorus and nitrogen concentrations in many parts of Lake Champlain, to levels that had not been recorded since 1990. During the spring, snowmelt combined with heavy rainfall increased the lake surface area by 106.2 km^2^ [133]. Agricultural lands were flooded for several weeks and sewage overflows were reported in both Quebec and Vermont [129,134]. The tropical storm Irene in August, produced 28 cm of rain within 24 h. It was associated with the input of close to 38 million liters of wastewater in the Vermont part of the MB watershed [129]. These important flood events may have considerably altered the eutrophication profile of MB and the nutrient limitation dynamics. In Quebec, severe cyanobacterial blooms have been reported in both 2011 and 2012. Several beaches were closed in 2012 especially on the Quebec side. It will be very interesting to see the long-term effects of these extreme events on the cyanobacterial diversity in the Missisquoi Bay/Pike River area and in Vermont. Recent calculations of nutrient loading into tributaries showed phosphorus concentrations 1.7 to 2.8 times above the average levels [129]. The nitrogen content in urban wastewater can also be as significant as phosphorus. The impact of the enhanced loading of nitrogen in Lake Champlain remains to be validated. A recent study by Beaulieu and colleagues [135] using a 1147 lakes data set has revealed that total nitrogen can become particularly relevant to predict cyanobacterial blooms when cyanobacteria dominate the phytoplankton community. Leavitt [136] demonstrated that nitrogen can enhance cyanobacterial growth and the production of toxins in eutrophic lakes. Other studies have shown that high nitrate concentrations in the water column may trigger the disappearance of *Microcystis aeruginosa* and favor the growth of other genera of cyanobacteria [99,137]. For example, *Dolichospermum* [85] is believed to possess several mechanisms for nitrogen competition [35]. Several members of this genus have the ability to produce microcystin, cylindrospermopsis and anatoxin-a.

## 5. Conclusions

Knowledge of the site-specific bloom-promoting factors combined with the identification of external sources of nutrients can contribute to the design of more effective management strategies for the abatement of toxic cyanobacterial blooms in this affected area. Significant challenges remain since both countries are managed under a different set of regulations. The resulting approaches and timelines to reduce phosphorus are different. Addressing nutrient overload will require the continuous cooperation and coordination at the provincial, federal, state and international levels. Corrective measures should target the most sensitive areas in both countries. Modifications should include the upgrade of drinking water production plants for health purposes, the upgrade of wastewater treatment facilities as well as the installation of storm water runoff systems. Changes should also include the implementation of agricultural practices that reduce erosion and limit surface runoff of phosphorus, nitrogen, total coliforms, *E. coli* and pathogens.
